# How polymorphic markers contribute to genetic diseases in different populations? The study of inhibin A for premature ovarian insufficiency

**DOI:** 10.1590/S1679-45082017AO4052

**Published:** 2017

**Authors:** Denise Maria Christofolini, Emerson Barchi Cordts, Fernando Santos-Pinheiro, Erika Azuma Kayaki, Mayla Cristina Fernandes Dornas, Monise de Castro Santos, Bianca Bianco, Caio Parente Barbosa

**Affiliations:** 1 Faculdade de Medicina do ABC, Santo André, SP, Brazil.

**Keywords:** Menopause, premature, Ovarian follicle, Mutation, Polymorphism, genetic, Inhibins, Menopausa precoce, Folículo ovariano, Mutação, Polimorfismo genético, Inibinas

## Abstract

**Objective:**

To verify the incidence of the G679A mutation in exon 2 of the *gene inhibin alpha* (*INHA*), in women with secondary amenorrhea and diagnosis of premature ovarian insufficiency, and in controls.

**Methods:**

A 5mL sample of peripheral blood was collected from all study participants in an EDTA tube and was used for DNA extraction. For the patient group, 5mL of blood were also collected in a tube containing heparin for karyotype, and 5mL were collected in a dry tube for follicle stimulant hormone dosage. All patient and control samples were initially submitted to analysis of the G679A variant in exon 2 of the *INHA* gene by PCR-RFLP technique. Samples from patients with premature ovarian insufficiency after PCR-RFLP were submitted to Sanger sequencing of the encoding exons 2 and 3. Sequencing was performed on ABI 3500 GeneticAnalyzer equipment and the results were evaluated by SeqA and Variant Reporter software.

**Results:**

Samples of 70 women with premature ovarian insufficiency and 97 fertile controls were evaluated. The G769A variant was found in only one patient in the Premature Ovarian Insufficiency Group and in no control, and it appears to be rare in Brazilian patients with premature ovarian insufficiency. This polymorphism was previously associated to premature ovarian insufficiency in several populations worldwide.

**Conclusion:**

There is genetic heterogeneity regarding the *INHA* gene in different populations, and among the causes of premature ovarian insufficiency.

## RESUMO

**Objetivo:**

Verificar a incidência da mutação G679A no éxon 2 do gene da *inibina alfa* (*INHA*) em mulheres com amenorreia secundária e diagnóstico de insuficiência ovariana prematura e em controles.

**Métodos:**

Uma amostra de 5mL de sangue periférico foi coletada de todos os participantes do estudo em tubo de EDTA e utilizada para a extração de DNA. Para o grupo de pacientes, foram coletados também 5mL de sangue em tubo contendo heparina para realização de cariótipo, e 5mL um tubo seco para dosagem de hormônio folículo-estimulante. As amostras de pacientes e controles foram inicialmente submetidas à análise da variante G679A no éxon 2 do gene INHA pela técnica de PCR-RFLP. As amostras de pacientes com insuficiência ovariana prematura após PCR-RFLP foram submetidas ao sequenciamento de Sanger dos éxons codantes 2 e 3. O sequenciamento foi realizado em equipamento ABI 3500 GeneticAnalyzer, e os resultados foram avaliados pelos programas SeqA and Variant Reporter.

**Resultados:**

Foram avaliadas amostras de 70 mulheres com insuficiência ovariana prematura e de 97 controles férteis. A variante G769A foi encontrada em apenas uma paciente do Grupo Insuficiência Ovariana Prematura e em nenhum controle, e parece ser rara nas pacientes brasileiras com insuficiência ovariana prematura. Este polimorfismo foi previamente associado à insuficiência ovariana prematura em diversas populações no mundo.

**Conclusão:**

O estudo evidenciou que há heterogeneidade genética quanto ao *INHA* em diferentes populações e entre as causas de insuficiência ovariana prematura.

## INTRODUCTION

Premature ovarian failure (POF), which has been recently called premature ovarian insufficiency (POI), is a process by which the gradual decline of ovarian function results in failure of folliculogenesis before 40 years of age. It is characterized by the absence of menstruation for a period longer than 4 months (secondary amenorrhea), but can be manifested before menarche, leading to amenorrhea.^(^ ^1^ ^)^ In 2015, the European Society of Human Reproduction and Embryology guidelines defined as criteria for POI the observation of two measurements of follicle stimulanting hormone (FSH) levels higher than 25IU (international units), performed within a 4-week interval.

The incidence of POI in patients with karyotype 46, XX is 1:1,000 up to 30 years of age, 1:250 at 35 years and 1:100 at 40 years.^(^ ^2^ ^)^ The prevalence of POI in patients with primary amenorrhea is 10 to 28%, and 4 to 18% with secondary amenorrhea.^(^ ^3^ ^,^ ^4^ ^)^ The causes of POI encompass a broad spectrum, ranging from autoimmune to iatrogenic conditions, but the sporadic form of POI is the most common.^(^ ^5^ ^)^ In about 5% of cases there is a positive family history, which suggests a genetic predisposition to the disease.^(^ ^6^ ^)^

Although they represent only a small fraction of cases, the X chromosome abnormalities (13%), autoimmunity (30 to 40%) and premutation of the *fragile mental retardation 1* (*FMR1*) gene (6%) have been associated with the incidence of POI.^(^ ^7^ ^)^ Moreover, the literature describes polymorphisms and mutations in several genes related to sporadic form of POI, including the *follicle stimulating hormone receptor* (*FSHR*), *luteinizing hormone receptor* (*LHR*) and *inhibin alfa (INHA).* ^(^ ^8^ ^-^ ^14^ ^)^

Shelling et al.^(^ ^15^ ^)^ established the first evidence of a genetic link between inhibin and POI, by the cytogenetic analysis of a POI patient who had the karyotype 46,XX,t(2;15)(q32.3;q13.3). The *INHA* gene is located at 2q33-36, and this rearrangement provided a clue that inhibin might play a role in the development of POI.

Inhibins are dimeric glycoproteins predominantly produced in the gonads.^(^ ^16^ ^)^ They act regulating FSH secretion in the normal menstrual cycle, process that allows a single programmed mature follicle ovulation.^(^ ^17^ ^)^ The inhibin subunits are encoded by three separate genes: *INHA, inhibin-beta A* (*INHBA*), and *inhibin-beta B* (*INHBB*), which were mapped to 2q33-qter, 2cen-q13, and 7p15-p14, respectively.

Previous studies^(^ ^15^ ^,^ ^18^ ^)^ suggested the involvement of the inhibin A in the etiology of POI, since defects on inhibin secretion may cause increased FSH concentration, follicle recruitment, alteration and premature depletion of the ovarian follicle pool.^(^ ^15^ ^)^ *In vitro* functional analysis^(^ ^19^ ^)^ provided evidence that G769A variant may increase susceptibility to POI with impaired INHBB bioactivity.

## OBJECTIVE

To study the incidence of G769A mutation in exon 2 of inhibin-alpha gene in women with secondary amenorrhea diagnosed with idiopathic premature ovarian insufficiency and controls.

## METHODS

Seventy patients diagnosed as POI were recruited from Human Reproduction and Genetics Center of *Faculdade de Medicina do ABC, Santo André, São Paulo,* from January 2008 to July 2016. They underwent a detailed medical history and gynecological evaluation. Clinical criteria for POI met ESHRE guidelines 2015. An important fact about the clinical history of these women with POI was complaint of infertility. As an inclusion criterion, all patients presented normal karyotypes.

The Control Group included 97 healthy women who had undergone physiological menopause after 48 years of age, fertile, with normal menstrual history, regular menses (duration 25 to 35 days), no personal or familial history of premature or early menopause, and no consumption of oral contraceptives or other hormone medications at the time of recruitment. The participation was voluntary and all women signed an Informed Consent form approved by the Ethics Committee of *Faculdade de Medicina do ABC*, under protocol number 184/2007.

All patients underwent a complete clinical examination, with a complete medical and gynecologic history, including the reproductive health of the patient’s mother, family history, consanguinity, and other genetic conditions in the family, age at menarche, and age at menopause.

A 5mL sample of peripheral blood from study participants was collected in an EDTA tube for DNA extraction, using the GE kit Spin Blood Mini Kit genomic Prep.

Patients and control samples were first submitted to restriction fragment length polymorphism obtained after polymerase chain reaction amplification (RFLP-PCR). Reactions were performed according to Jeong et al.,^(^ ^20^ ^)^ with 100ng of DNA, primers (5nM; forward: 5’GGCCCACACTCGGACCAGAC3’, reverse: 5’ AGCCCACAACCACCATGACAGTAG 3’), 10% of PCR buffer, 50nmol dNTPs (deoxynucleotide triphosphates) and 1U (unit) Taq DNA polymerase in final volume of 50mL. Polymerase chain reaction conditions were: denaturation at 94°C for 5 minutes, 30 cycles (45 seconds at 94°C, 45 seconds at 65°C, 1 minute at 72°C) and 10-minute extension at 72°C. For enzyme digestion 8uL of sample, 2U of restriction enzyme BbvI, 1x buffer and sterile water were used. The mixture was incubated at 37°C for 3 hours, and inactivated at 65°C for 20 minutes, and subjected to electrophoresis on 3% agarose gel. After digestion, the mutant allele present a fragment of 203 base pair (bp) and normal allele present two fragments, one with 85 and another of 159 bp. The heterozygous presented three fragments: 203, 159 and 85 bp.

After RFLP analysis, we decided to perform Sanger sequencing of *INHA* coding exons 2 and 3. They were amplified by PCR, from 100ng of DNA sample in the presence of 5nmol of each primer (forward: 5’ GCCCACACTCGGACCAGAC 3’; reverse 5’ CGTGAGAAGGTTGGGCACTG 3’), purified by STRATEC kit and prepared for sequencing using BigDye^®^ Terminator v3.1 Cycle Sequencing Kit, according to manufacturer protocol (AppliedBiosystems, Foster City, CA, USA). Sequencing was performed in an ABI 3500 GeneticAnalyzer and results were evaluated by SeqA and VariantReporter software. Statistical analysis was performed by Stata 11 software. Fisher exact test was performed to compare the incidence of genotypes and alleles among case and control samples.

## RESULTS

Seventy women with confirmed idiopathic POI were investigated, with mean age of 36.0 years (±7.49 years). As to the Control Group, the mean age was 48 years. The mean age of last menstrual period in patients with POI was 31.5 years (±6.59 years). The symptoms most commonly reported during the medical visits were hot flashes, infertility, decreased libido and atrophy of the genitourinary tract. The mean level of FSH was 64.3mIU/mL. According to RFLP-PCR and sequencing analysis of the samples only one patient showed to be heterozygous for the G769A mutation (Table 1).

**Table 1:** Genotype and allelic distribution for G769A polymorphism in Brazilian patients with premature ovarian insufficiency and controls

Group	Genotypes				Allelic distribution		
	
	GG	GA	AA	p value	G	A	p value
POI	69	1*	0	0.416	139	1	0.419
Control	97	0	0		194	0	

* The frequency of the A allele in our Brazilian sample was 0.003.

POI: premature ovarian insufficiency.

## DISCUSSION

The impaired production of inhibin has been associated with natural menopause and the development of POI. Moreover, high levels of inhibin A in women with POI are similar to those observed in postmenopausal women.^(^ ^21^ ^)^ Several studies suggest that decreased levels of inhibin during the perimenopausal women, associated with the concomitant increased levels of activin A, may be responsible for high FSH levels, which are characteristic of reproductive function aging. Although the reduced inhibin/activin ratio observed during menopause is probably due to impaired synthesis of inhibin,^(^ ^22^ ^)^ ovarian failure might be thought to result from mutations in *INHA* gene, which would lead to decreased inhibin concentration and, consequently, increased FSH levels.

The presence of the G769A substitution in exon 2 of the *INHA* gene was first studied by Shelling et al.,^(^ ^15^ ^)^ in three of 43 women with POI compared with one of 150 controls. Later, Marozzi et al.,^(^ ^18^ ^)^ reported that the G769A transition was significantly more frequent in patients with POI (7 of 157) than in the Control Group (zero of 100). The hypothesis raised is that this substitution would hinder the affinity of inhibin for its receptor.^(^ ^15^ ^)^ This mutation was also detected in 9 of 80 patients with POI, and zero of 100 controls in India.^(^ ^23^ ^)^ Prakash et al.,^(^ ^24^ ^)^ found this mutation in 13 of 100 Indian women with POI, and in 2 of 50 controls. They also found three new variants in *INHA* gene in one POI patient. However, a Korean study did not demonstrate G769A substitution in any of the 84 patients with POI nor in 100 controls.^(^ ^20^ ^)^ Yet, the functional significance of the amino acid variant at codon 257 is still unknown, since no functional study has been carried out so far. Another study on the G769A transition was conducted by Sundblad et al.,^(^ ^25^ ^)^ in Argentina, and found no association between G769A mutation and POI, when evaluating 59 patients and 73 controls.

Other mutations on the *INHA* gene were also associated to POI. The studies by Harris et al.,^(^ ^26^ ^)^ Woad et al.,^(^ ^27^ ^)^ and Dixit et al.,^(^ ^28^ ^)^ with populations of New Zealand, Slovenia and India, observed significant differences in allelic frequency in *INHA* promoter among POI groups and controls, and concluded that such variations were related to manifestation of POI. In order to shed some light to the contradictory results of association between *INHA* polymorphisms and POI, Zintzaras et al.,^(^ ^29^ ^)^ performed a meta-analysis of *INHA* G769A, C16T, A124G mutations and their association to POI. Considering the cumulative data, none of the mutations provided association to POI. The association was only indicated of *INHA* G769A mutation in Asian Indians.

The present study evaluated a group of 70 women with Premature Ovarian Insufficiency Group and Control Group composed by 97 women over 40 years and with normal menstrual cycles. The substitution G769A in exon 2 of *INHA* gene was found in one patient, but in no controls (allelic frequency of 0.003). No statistical difference between the groups was observed. This result corroborates the findings of Sundblad et al.,^(^ ^25^ ^)^ and Jeong et al.,^(^ ^20^ ^)^ and suggests that the exchange G769A is uncommon in Brazilian women with POI.

One limitation of the present study is the sample size. This was due to the fact that the event is rarely perceived, since many women at child-bearing age take oral contraceptives that “mask” irregular periods. Moreover, our sample was rigorously selected, being free of abnormal karyotype and *FMR1* pre-mutation. The sample is also larger than most of POI published articles. Notwithstanding this limitation, this study provides evidence to show genetic heterogeneity in the *INHA* gene in different populations and in the etiology of POI.

Premature ovarian insufficiency is a complex character condition, determined by interactions between genetic and environmental factors, and it would be of great interest to characterize the actual relation between the inhibins and POI in a large number of cases.

## CONCLUSION

The study showed that there is genetic heterogeneity regarding the *INHA* gene in different populations and among the causes of premature ovarian insufficiency.
